# Surgical Emphysema Post Liposuction Overseas: A Case Report

**DOI:** 10.1093/asjof/ojaf085

**Published:** 2025-07-28

**Authors:** Hannah Cook, Samim Ghorbanian, Marios Erotocritou, Lucy Coull, Yildirim Oezdogan

## Abstract

Liposuction is the most common surgical procedure performed globally. It is generally associated with low rates of major complications. Cosmetic surgery tourism has seen a steep incline in popularity and is often favorable to patients because of lower costs. However, it can be associated with higher complication rates and poorer follow-up. In this case report, the authors describe a case of severe symptomatic subcutaneous emphysema following air travel post liposuction. A 49-year-old male presented to the emergency department in the United Kingdom with pain, bruising, and swelling post liposuction to the chest, abdomen, and flanks. At Day 5 postoperatively, the patient embarked on a 5-hour plane journey, during which they experienced a significant increase in pain and swelling. On examination, there was audible crepitus at the upper abdomen and chest. A computerized tomography scan revealed extensive subcutaneous emphysema with associated skin thickening, subcutaneous fat stranding, and fluid. In this case, subcutaneous emphysema is likely a direct result of surgical technique. This case is unique in the severity of the symptoms described and the presumed role of air travel in their exacerbation. It is important that patients are adequately counseled on risks that may arise because of flying in the close postoperative period. Another issue highlighted is the increasing number of presentations to UK public hospitals following cosmetic tourism. Subcutaneous emphysema is a recognized but rare complication of liposuction. Patients should be adequately informed of adverse events that can arise following cosmetic surgery and subsequent travel.

**Level of Evidence: 4** (Therapeutic): 
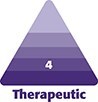

Liposuction is the most common surgical procedure performed globally, with 2.2 million procedures performed in 2023.^1^ The rate of major complications associated with liposuction is low, and it is generally seen as a safe procedure.^2^ Major complications associated with liposuction include hematoma, thromboembolism, and infection.^2^

Cosmetic surgical tourism, whereby a patient undergoes cosmetic surgery outside of the country they reside in, has seen a steep incline in popularity in recent years, in part because of associated lower costs when compared with cosmetic surgery in the United Kingdom.^3^ Despite this, cosmetic surgery abroad can be associated with higher complication rates.^4^ Because patients leave the country shortly following the procedure, adequate follow-up is often not available, and complications may arise upon return to the patient's home country.^5^

This case report describes subcutaneous emphysema arising following liposuction performed out of the patient's country of residence, likely exacerbated by pressure changes during air travel.

## CASE PRESENTATION

A 49-year-old gentleman presented to the emergency department in the United Kingdom with pain, bruising, and swelling 6 days post liposuction to the chest, abdomen, and flanks; the procedure was performed in Egypt. The operating surgeon performed ultrasound-assisted (VASER, Sound Surgical Technologies) and power-assisted (PAL, MicroAir, Charlottesville, VA) liposuction under general anesthetic, harvesting 8 L of fat. Four drains (bilateral chest and groin) were placed intraoperatively and removed at Day 3 postoperatively, with dressings placed over drain sites. Before discharge, the patient was given co-amoxiclav tablets, which were taken regularly, alongside a compression garment, which was not worn. According to the patient and surgeon, postoperative review was unremarkable; the patient was well and not in pain on departure from hospital.

At Day 5 postoperatively, the patient embarked on a 5-hour plane journey back to the United Kingdom, during which a significant increase in pain and swelling was noticed in the lower abdomen and flanks. The pain was so severe that it prohibited the patient from sitting down.

**Figure 1.**
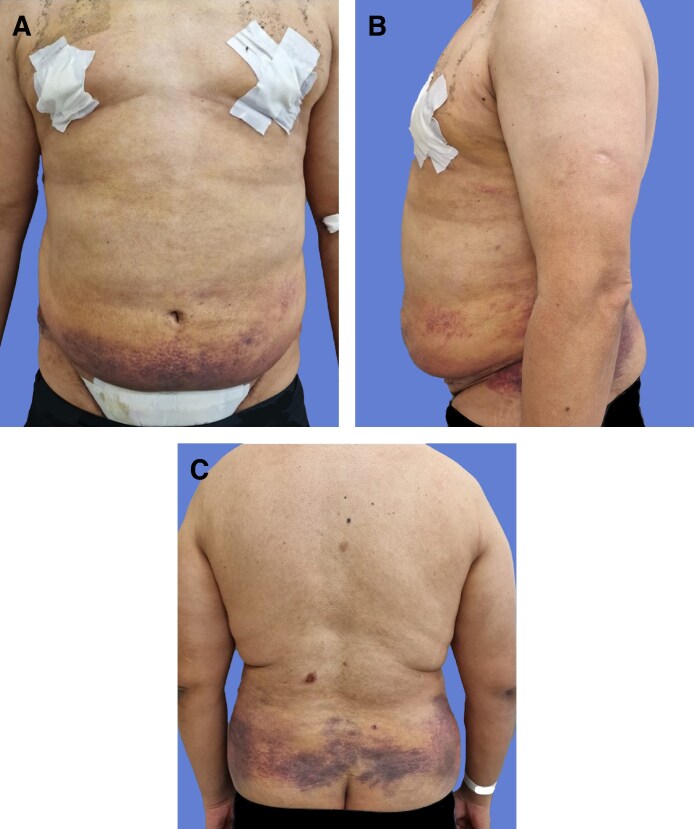
Clinical photographs of a 49-year-old male, 6 days post liposuction to the abdomen and flanks, performed out of country. The patient presented to the emergency department following air travel with subcutaneous emphysema to the chest, abdomen, and flanks. Note dressings over previous drain sites: (A) front; (B) side; (C) back.

On arrival to the United Kingdom (Day 6 postoperatively), the patient traveled straight to the emergency department where they were assessed by a member of the plastic surgery team. On examination, abdominal distension was present, with diffuse ecchymosis over the lower abdomen and bilateral flanks (Figure 1).

The patient had generalized tenderness without evidence of peritonitis. Subcutaneous emphysema was palpable, with audible crepitus at the upper abdomen, overlying the sternum and pectoral muscles. Fortunately, there was no clinical evidence of a necrotizing soft tissue infection.

**Figure 2.**
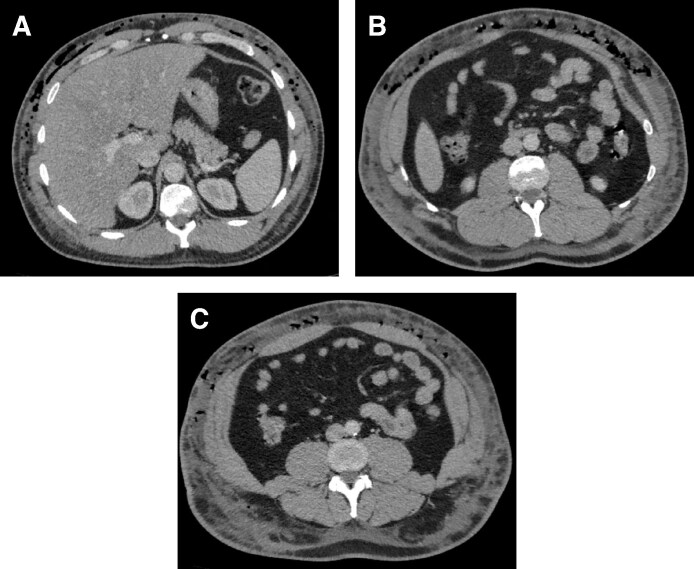
(A, B, C) Computerized tomography images of the abdomen performed at Day 6 postoperatively. Note extensive subcutaneous emphysema throughout the anterior abdomen and flanks, without evidence of free air within the peritoneal cavity.

Bedside observations revealed a tachycardia, but the patient remained normotensive and normothermic. BMI was 32 (weight 104 kg). Blood tests demonstrated mildly raised white cells and C-reactive protein; lactate and lipase were normal. A chest X-ray did not show free gas under the diaphragm. A subsequent contrast computerized tomography (CT) scan of the abdomen and pelvis was performed. This revealed extensive subcutaneous emphysema with associated skin thickening, subcutaneous fat stranding, and fluid. These inflammatory changes extended from the diaphragm into the flanks and lumbar region but not intraperitoneally. There was no visualized drainable collection. CT images are displayed in Figure 2.

A decision was made by the plastic surgery team that no surgical intervention was required at the time of review. The patient was discharged with analgesia and a 48 h follow-up appointment, with advice to re-present should their symptoms deteriorate in the meantime. When reviewed in outpatient clinic, the patient was significantly more comfortable. On examination, the bruising was not more extensive and had matured. Subcutaneous emphysema was still palpable but less diffuse. On telephone review at 4 weeks postoperatively, the patient remained well, with complete resolution of symptoms.

## DISCUSSION

This case describes an occurrence of severe postoperative pain associated with liposuction, likely exacerbated by air travel within 1 week of surgery.

Known complications associated with liposuction include iatrogenic perforation of the bowel as a result of the catheter being placed too deep, as well as severe necrotizing skin and soft tissue infections.^6^ These are well described in literature.^7^

The infiltration of air into subcutaneous tissues can occur following surgery, trauma, infection, or spontaneously.^8^ In this case, subcutaneous emphysema was caused not as sequelae of complications such as necrotizing fasciitis, but likely as a direct outcome of surgical technique. This has been sparsely reported in literature—Kim et al reported a similar presentation where the surgical emphysema was thought to involve the check valve, with subsequent accumulation of air in the subcutaneous tissue along a negative pressure gradient after entering through skin incisions.^9^ Lim et al report a presentation of subcutaneous emphysema in a patient who underwent liposuction alongside J-Plasma therapy (Renuvion, Clearwater, FL; helium plasma)—the latter of which was thought to result in this complication.^10,11^ Likewise in this case, power-assisted liposuction was used, which may have contributed to the severity of symptoms.

Fortunately, the symptoms in this case resolved with conservative measures. Compression garments are thought to reduce postoperative swelling and discomfort but there is little evidence for their role in reducing the incidence of complications such as seroma or hematoma.^12^ The majority of cases of subcutaneous emphysema are self-limiting once the cause is identified and resolved.^13^ For patients experiencing severe discomfort, high-concentration oxygen therapy has been employed to increase the rate of absorption of subcutaneous emphysema.^14^ Complications include restricted chest expansion and respiratory compromise (if in the chest) and genital skin necrosis (if disruption to cutaneous vasculature occurs).^13,15^ Alongside emergency resuscitation and multidisciplinary team involvement if required, some papers have suggested decompression through targeted incisions in restricted chest expansion.^13^ One case report described successful resolution of complications following insertion of a low-suction subcutaneous drain.^16^

This case is unique in that the described symptoms likely arose or were exacerbated following air travel. The presumed mechanism for this is the physiological effects of pressure changes caused by altitude and air travel. Reduced cabin pressures during ascent lead to an increase of gas volume expansion by ∼30%.^17^ Within the subcutaneous tissue, trapped gas would be unable to flow freely, prohibiting equalization of air pressure and causing pain. Such phenomena have been described among patients with lung disease and can have severe consequences, such as ruptured pulmonary bullae or decompensated closed pneumothoraces.^17^ However, as far as the authors are aware, this is the first time such effects have been described following liposuction.

This case highlights some key issues. Firstly, the increased risk of postsurgical complications following air travel. Literature lacks precise evidence and guidelines regarding air travel post surgery.^18^ The UK Civil Aviation Authority recommends avoiding flying for 5 to 10 days following “abdominal surgery,” whereas the American Society of Plastic Surgeons recommends waiting 5 to 7 days before air travel after liposuction.^19,20^ Although the patient flew at Day 5 postoperatively, their symptoms were severe enough to warrant a hospital visit, although fortunately there was no physiological compromise requiring intervention. It is important that patients are adequately and specifically counseled in risks that may arise as a result of flying in the close postoperative period. The second issue highlighted is the increasing number of patients presenting to UK public hospitals following surgery out of their country of residence to undergo cosmetic procedures.^21^ Aside from the cost incurred by the healthcare system of such presentations, there is also a detrimental impact on patients' health and can cause significant morbidity and mortality.^21^ The volume of fat routinely harvested during liposuction may also vary between countries; the American Society of Plastic Surgeons recommends 5 L as a safe limit, above which there is an increased risk of complications.^22^ In this case, 8 L was harvested; although large-volume liposuction may be performed safely by experienced surgeons in appropriately selected patients, variations in practice standards and regulation between countries may contribute to differing thresholds for acceptable volumes.^22^ When combined with factors such as long-distance travel, limited postoperative follow-up, and variations in perioperative support, this may increase the risk of complications. Global governing bodies should consider the merit of improved patient education and legislation to regulate practice in this sector.

A limitation of this report is that although symptoms were likely exacerbated by air travel because of their timing and severity, this is only 1 case, and other potential confounders cannot be adjusted for. In this case, noncompliance with compression garments is a potential factor to consider; however, gas expansion may well still have occurred underneath the garment.

## CONCLUSIONS

Subcutaneous emphysema is a recognized but rare complication of liposuction, and this reports the importance of recognizing it as a differential diagnosis to allow prompt management. Clearly, patients should be adequately informed during the consent process as to adverse events that can arise following cosmetic surgery, including specific travel-related complications when relevant.
